# Serotonin homeostasis and serotonin receptors as actors of cortical construction: special attention to the 5-HT_3A_ and 5-HT_6_ receptor subtypes

**DOI:** 10.3389/fncel.2013.00093

**Published:** 2013-06-19

**Authors:** Tania Vitalis, Mark S. Ansorge, Alexandre G. Dayer

**Affiliations:** ^1^Laboratoire de Neurobiologie, ESPCI ParisTech, Centre National de la Recherche Scientifique-UMR 7637Paris, France; ^2^Divisions of Developmental Neuroscience, Department of Psychiatry, Columbia UniversityNew York, NY, USA; ^3^Department of Mental Health and Psychiatry, University Hospital of GenevaGeneva, Switzerland; ^4^Department of Basic Neurosciences, University of Geneva Medical SchoolGeneva, Switzerland

**Keywords:** 5-HT, somatosensory cortex, cerebral cortex, development, plasticity, 5-HT3 receptor, 5-HT6 receptor, circuit assembly

## Abstract

Cortical circuits control higher-order cognitive processes and their function is highly dependent on their structure that emerges during development. The construction of cortical circuits involves the coordinated interplay between different types of cellular processes such as proliferation, migration, and differentiation of neural and glial cell subtypes. Among the multiple factors that regulate the assembly of cortical circuits, 5-HT is an important developmental signal that impacts on a broad diversity of cellular processes. 5-HT is detected at the onset of embryonic telencephalic formation and a variety of serotonergic receptors are dynamically expressed in the embryonic developing cortex in a region and cell-type specific manner. Among these receptors, the ionotropic 5-HT_3A_ receptor and the metabotropic 5-HT_6_ receptor have recently been identified as novel serotonergic targets regulating different aspects of cortical construction including neuronal migration and dendritic differentiation. In this review, we focus on the developmental impact of serotonergic systems on the construction of cortical circuits and discuss their potential role in programming risk for human psychiatric disorders.

## Introduction

The mammalian cerebral cortex is critical for sensory-motor integration, higher-order cognitive functions, and emotional regulation. It processes information through the activation of neural networks composed of excitatory glutamatergic pyramidal neurons and local modulatory interneurons that release γ-aminobutyric acid (GABA), neuropeptides, and vasoactive substances (Peters and Jones, 1984; Peters and Kara, 1985a,b; Baraban and Tallent, 2004; Karagiannis et al., 2009; Tricoire and Vitalis, 2012). Developmental perturbations impacting the maturation of cortical circuits can confer risk for neuropsychiatric disorders (Insel, 2010; Thompson and Levitt, 2010; Marin, 2012). Our labs have contributed to a model, in which such developmental vulnerability is often restricted to sensitive periods. The concept of sensitive developmental periods for the indelible modulation of complex behaviors is similar to that described for sensory systems (i.e., visual cortex, ocular dominance plasticity), but modulating factors, and underlying mechanisms are much less well-understood.

Building cortical circuits relies on a series of precisely timed events that take place mainly during embryonic and early postnatal development (reviewed in Marin and Rubenstein, 2001; Bystron et al., 2008; Corbin et al., 2008; Batista-Brito and Fishell, 2009; Rakic, 2009; Vitalis and Rossier, 2011). Critical components include the proliferation, migration, and differentiation of neurons and glial cells, with differentiation including the appropriate growth and guidance of axons toward their targets. These steps are genetically programmed and phylogenetically conserved, yet they are malleable and plastic. As cell-autonomous signaling unfolds over time, the various cortical cell-types are continuously in contact with and responding to their environment. Cell extrinsic signals are very diverse in nature and include monoamines, guidance cues, growth factors, cell adhesion molecules, and various components of the extracellular matrix. In particular, the monoamine 5-HT has emerged as an important regulator of neural circuit formation (previously reviewed in Gaspar et al., 2003; Vitalis and Parnavelas, 2003).

In developing rodent embryos, cortical 5-HT mainly arises from placental sources at the onset of cortical development and from serotonergic afferents by E16–E17 (Bonnin et al., 2011). This dual source of 5-HT is conserved in humans and permits 5-HT signaling during development, even before embryonic serotonergic neurons have differentiated and are able to release 5-HT. Not surprisingly, 5-HT modulates neuronal proliferation, migration, and differentiation, and is implicated in the etiology of many neuropsychiatric disorders, including mental retardation, autism, depression, and anxiety (for reviews, see Berger-Sweeney and Hohmann, 1997; Levitt et al., 1997; Whitaker-Azmitia, 2001; Gu, 2002; Gaspar et al., 2003; Homberg et al., 2009; Oberlander et al., 2009; Daubert and Condron, 2010; Lesch and Waider, 2012). In the context of developmental plasticity under normal conditions as well as in disease, it is important to appreciate that 5-HT signaling is influenced by many factors, including nutrition (Serfaty et al., 2008), perinatal stress (Peters, 1990; Papaioannou et al., 2002a,b), infection (Winter et al., 2008, 2009), 5-HT metabolism and storage (Cases et al., 1996; Vitalis et al., 1998, 2007; Noorlander et al., 2008; Popa et al., 2008), genetic alterations (Lira et al., 2003; Murphy and Lesch, 2008; Pluess et al., 2010; Karg et al., 2011; Bonnin et al., 2011), and pharmacological compounds such as selective 5-HT reuptake inhibitors (Ansorge et al., 2004, 2008).

Here we review findings demonstrating that early-life 5-HT signaling regulates cellular events implicated in the assembly of cortical circuits. We highlight recent studies that have revealed the role of specific 5-HT receptors in the construction of such circuits: the ionotropic 5-HT type 3A receptor (5-HT_3A_) and the metabotropic 5-HT type 6 receptor (5-HT_6_). Finally, we review clinical studies suggesting that altered 5-HT homeostasis or signaling could increase risk for human stress-related psychopathologies such as mood and anxiety disorders.

## Structure and development of the rodent cerebral cortex

### Neuronal components

The cerebral cortex of adult mammals is a laminated structure comprised of six layers that each contain a complement of pyramidal (glutamatergic) and non-pyramidal (GABAergic) neurons (Peters and Jones, 1984). Pyramidal neurons make up ~80% of all adult cortical neurons, sending excitatory output axons to other cortical areas and to distant parts of the brain (Peters and Kara, 1985a; Thomson and Lamy, 2007; Spruston, 2008). The vast majority of cortical GABAergic cells are interneurons that only make local connections. GABAergic interneurons are extremely diverse, differing in shape, electrophysiological properties, and the combination of neuropeptides and calcium-binding proteins that they express (Peters and Kara, 1985b; Cavanagh and Parnavelas, 1988; DeFelipe, 1993; Kawaguchi and Kondo, 2002; Blatow et al., 2005; Tomson and Lamy, 2007; PING et al., 2008; Karagiannis et al., 2009; Xu et al., 2010; Vitalis and Rossier, 2011; Tricoire and Vitalis, 2012; DeFelipe et al., 2013). Using these differentiating characteristics, one can at a first approximation distinguish four main classes of interneurons populating the somatosensory cortex (PING et al., 2008; DeFelipe et al., 2013). First, fast-spiking interneurons that express parvalbumin (Parv), and act as an inhibitory gate for incoming sensory information (Inoue and Imoto, 2006; Sun et al., 2006). Second, adapting martinotti cells that express somatostatin (SOM), and are thought to control dendritic information through local feedback inhibition (Karube et al., 2004). Third, adapting bipolar interneurons that express vasoactive intestinal peptide (VIP) and calretinin (CR), and preferentially target other interneurons and receive direct input from the thalamus (Férézou et al., 2007; Vitalis and Rossier, 2011). Fourth, adapting neurogliaform interneurons that express neuropeptide Y (NPY) and/or nitric oxide (NO), and that are responsible for the slow GABAergic inhibition of pyramidal cells and interneurons (Karagiannis et al., 2009; Oláh et al., 2009; Perrenoud et al., 2012a,b; Tricoire and Vitalis, 2012).

### Development of the cerebral cortex

#### Origins and migration of pyramidal neurons and the formation of cortical layers

The cerebral cortex develops from neuroepithelial germinal cells of the telencephalic pallium and subpallium that massively proliferate (from E11 to E12 in mice), to form the cerebral vesicles. The first neurons generated, Cajal-Retzius (C-R) cells and subplate (SP) cells, form transient and heterogeneous populations of cells that originate from both pallial and subpallial territories and establish the preplate (PP; Boulder Committee, 1970; Uylings et al., 1990; Bystron et al., 2008). SP and reelin secreting C-R cells provide positioning cues and instructions to developing cortical neurons and afferents (Supèr et al., 2000; Soriano and del Rio, 2005; Herz and Chen, 2006; Lakatosova and Ostatnikova, 2012). The first pyramidal neurons generated arise sequentially from the cortical ventricular zone (VZ), from which they translocate or migrate radially to form a layer within the PP, the so-called cortical plate (CP), thus splitting the PP into a superficial marginal zone (MZ; presumptive layer I containing the C-R cells) and a deep SP. At the beginning of CP formation (E13–E14 in mice), pyramidal cells are generated from radial glial cells (RGCs), whereas later (E15–E17 in mice), they mainly originate from intermediate progenitor cells (IPC; or basal progenitors) deriving from RGC cells (see Kriegstein and Noctor, 2004; Noctor et al., 2004; Corbin et al., 2008 for reviews). The neurons of the CP assemble into layers II–VI in an “inside-out” sequence: the deepest cellular layers are assembled first and those closest to the surface last.

#### Origins and migration of GABAergic neurons

In rodents, most GABAergic neurons are generated outside the cortical VZ, mainly in the medial (E11–E14 in mice) and the caudal (E14–E17 in mice) parts of the ganglionic eminence (MGE and CGE, respectively) in the basal telencephalon (for reviews, see Marin and Rubenstein, 2001; Wonders and Anderson, 2006; Batista-Brito and Fishell, 2009; Rudy et al., 2011; Vitalis and Rossier, 2011), and more ventrally in the entopeduncular region (AEP) and the preoptic region (POA; Gelman et al., 2009). These areas are specified through a combination of distinct transcription factors and morphogenes, and produce different classes of interneurons. The ventral and dorsal parts of the MGE express the homeobox transcription factor Lhx6 and generate two large classes of interneurons: fast-spiking/Parv+ interneurons and adapting/SOM+ interneurons (Xu et al., 2004; Butt et al., 2005, 2007; Miyoshi et al., 2007; Wonders et al., 2008). Later, the CGE—a region that expresses the transcription factor Gsh2 (Fogarty et al., 2007) but lacks the transcription factors Nkx2.1, Nkx6.2, and Lhx6 (Flames et al., 2007)—generates an average of 30% of the total population of GABAergic interneurons, which mainly express VIP, CR, and NPY (Lee et al., 2010; Vucurovic et al., 2010; Rudy et al., 2011; Vitalis and Rossier, 2011). Once produced, interneurons migrate toward the CP. They initially follow parallel migratory streams, first in the intermediate zone and MZ, and later along the subventricular zone (SVZ), before they switch their migratory mode and incorporate into the developing CP through radial migration. Interestingly, some of the later generated mainly CGE-derived interneurons pause longer (until around P1–P2) in the SVZ before entering CP. In mice, cortical migration is almost completed by P4, and followed by cortical expansion. However, during the first postnatal days and decreasing with age the SVZ retains the capacity to produce CR-expressing interneurons that incorporate into the cerebral cortex at postnatal stages (Inta et al., 2008; Riccio et al., 2012). These key events are recapitulated in Figure 2.

## Sources of 5-HT to the rodent cortex

### 5-HT synthesis

5-HT is synthesized from the essential amino-acid tryptophan. In the blood stream, 90% of tryptophan is linked to serum-albumin. A proportion reaching ~10% when the blood-brain barrier becomes fully functional (postnatal day 12) and decreasing with age is free to cross the developing blood-brain barrier (Ribatti et al., 2006). Tryptophan is accumulated in 5-HT producing cells by a non-specific transporter with high affinity to several uncharged aromatic amino-acids. Tryptophan is then hydroxylated in these cells into 5-hydroxytryptophan by the tryptophan hydroxylase. Tryptophan hydroxylase type 2 (Tph2) is expressed in serotonergic neurons of the raphe nuclei (Côté et al., 2003; Walther et al., 2003), while peripheral tissues mostly express tryptophan hydroxylase type 1 (Tph1). 5-hydroxytryptophan is then further decarboxylated into 5-HT by the aromatic amino-acid decarboxylase (AADC). 5-HT is catabolized in the cytoplasm of 5-HT transporter (SERT) expressing cells by monoamine oxidase A or B (MAOA or MAOB). MAOA has higher affinity to 5-HT than MAOB, but both enzymes are co-expressed in rodent serotonergic neurons between E12 and P7 (Vitalis et al., 2002a). After P7, the expression of MAOB becomes predominant, and MAOA deficiency could be partially compensated for by the increased expression of MAOB in serotonergic neurons (Cases et al., 1996; Vitalis et al., 2002a; Cheng et al., 2010).

5-HT of the embryonic telencephalon is not only produced locally by serotonergic neurons of the raphe nuclei, but also originates from extra-CNS (embryonic periphery, placental) as well as extra-embryonic (maternal) sources. In the two following sections, we briefly recapitulate the development of the serotonergic system and review the various sources of telencephalic 5-HT during embryonic and early postnatal life.

### Development of the serotonergic system in relation to telencephalic development

**Figure 1:**
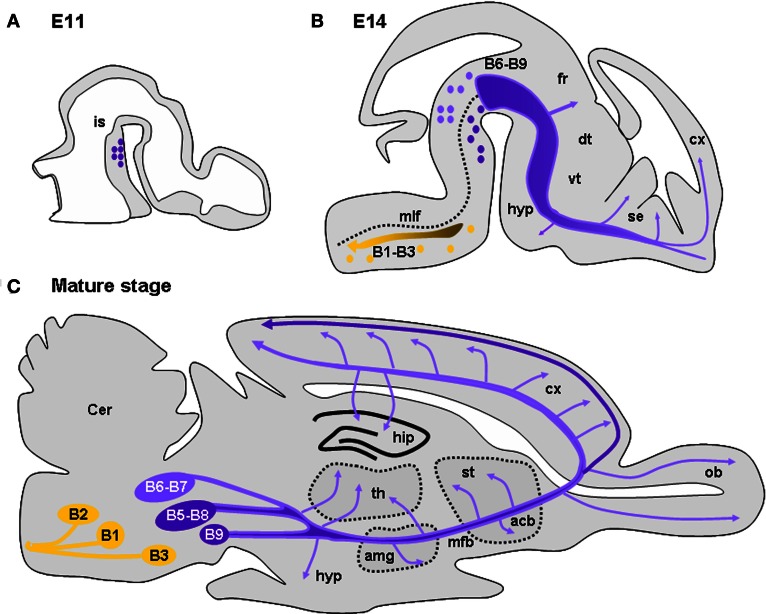
**Development and location of serotonergic neurons and projections. (A)** Drawing of a sagittal section of an E11 mouse brain illustrating the location of 5-HT neurons caudal to the isthmus (is). **(B)** Drawing of a sagittal section of an E15 mouse brain showing the 5-HT-containing cell groups and their main projections. 5-HT cell groups are classically distributed in nine groups (B1–B9). The posterior groups (B1–B3; yellow dots) mainly consist in raphe magnus sending projections to the medulla, the spinal cord, and the periphery (yellow dots and arrow). The raphe dorsal (B6, B7) located dorsal to the medial longitudinal fasiculus (mlf) and the raphe median (B5, B8) send ascending serotonergic innervations destine to the telencephalon and diencephalon (purple). Serotonergic afferents innervating the forebrain travel initially together along the median forebrain bundle (mfb). Arrows indicate regions where axons are seen to deviate from the major ascending pathway: along the fasciculus retroflexus (fr) toward the habenula, in the hypothalamus (hyp), the striatum, and the septum (se). **(C)** Drawing of a sagittal section of a mature mouse brain showing the 5-HT-containing cell groups and their main projections. Arrows indicate regions highly innervated by 5-HT afferents: the hypothalamus (hyp), the amygdala (amg), the thalamus (th), the accumbens (acc), the striatum (st), the olfactory bulb (ob), the cerebral cortex (cx), and the hippothalamus (hip). In the cerebral cortex, layers V and VI receive preferentially afferents arising from the dorsal raphe (light purple) while layer I receives afferents mainly arising from the median raphe (dark purple). Drawings are adapted from Wallace and Lauder (1983) and Steinbusch and Nieuwenhuys (1983).

Serotonergic neurons of the brainstem are subdivided into 9 groups forming two clusters: the caudal division (B1–B4; including the raphe pallidus, obscurus, magnus, and pontis) projecting to the spinal cord and the cerebellum, and the rostral division [B5–B9; including the dorsal (B6, B7) and median raphe nuclei (B5, B8)] projecting to the forebrain (Lidov and Molliver, 1982; Steinbusch and Nieuwenhuys, 1983; Törk, 1990; Figure 1A). Recent genetic and developmental approaches revealed differential rhombomeric identities of raphe 5-HT neurons, which introduce a new layer of functional classification (Jensen et al., 2008; Kiyasova and Gaspar, 2011). Together with genetic tracing and topographic projection mapping, we will soon have a much better understanding of the anatomical organization of the 5-HT systems.

In mice, dorsal raphe neurons differentiate in the brainstem by E10–E11 (E12–E15 in rats). This period coincides with the beginning of telencephalic vesicle formation (Wallace and Lauder, 1983; Aitken and Törk, 1988). Serotonergic neurons generated rostral to the isthmus (B6–B9 groups; dorsal and median raphe) send axons only one day after their genesis. These axons reach the cortico-striatal junction by E14 in mice (by E16 in rats; Wallace and Lauder, 1983; Figure 1B), during the peak of migration of cortical GABAergic interneurons generated in the MGE. 5-HT-containing axons enter the cortical anlage as two tangential streams, one above and the other below the CP (Wallace and Lauder, 1983; Aitken and Törk, 1988). The former is distributed in the MZ where pioneering C-R cells are located and with which they are in close appositions, making transient synaptic contacts (Radnikow et al., 2002; Janusonis et al., 2004).

Below the CP, 5-HT afferents are mainly restricted to the IZ and the SP (Wallace and Lauder, 1983). At E14, the developing cerebral cortex (Bayer and Altman, 1991) and the ganglionic eminences produce deeper-layer neurons (glutamatergic and GABAergic, respectively) that are in the process of migration to their final positions. By E16–E17 in mice, thalamocortical axons (TCAs) penetrate the cortical anlage and are in close apposition with 5-HT axons running in the IZ. In parallel, cortical neurons begin to establish their polarity, sending their axons toward their respective targets and developing numerous dendritic processes. At the end of corticogenesis, 5-HT axons gradually arborize sending numerous branches into the CP (Wallace and Lauder, 1983). During this period a large proportion of GABAergic interneurons enter the CP where they radially migrate to reach their final positions (see above). Progressively, serotonergic axons become evenly distributed in the different cortical territories and show their mature pattern of innervation by P21 (Steinbusch, 1981). However, dorsal raphe and median raphe projections differ anatomically. The dorsal raphe projections have been described as generally thin, displaying numerous branches with pleiotropic varicosities and preferentially arborize in cortical layers IV and V that receive thalamic inputs. By contrast, median raphe projections are characterized by large spherical varicosities that can form true chemical synapses (Törk, 1990). They preferentially arborize in layer I and lower white matter, give collaterals that could surround neuronal cell bodies and proximal dendrites, and preferentially contact interneurons containing VIP- and cholecystokinin (CCK) in various species (Törk, 1990; Hornung and Celio, 1992; Férézou et al., 2007). Interestingly, in the mature brain, a dense plexus of 5-HT-positive fibers is present in the SVZ, in close apposition with progenitor cells of this region (Jahanshahi et al., 2011). At all stages 5-HT could be released along the entire axonal network thus diffusing into the entire extracellular fluid. It is still not clear whether subsets of serotonergic axons preferentially release 5-HT in synaptic clefts vs. volume transmission.

### Other sources of 5-HT

**Figure 2:**
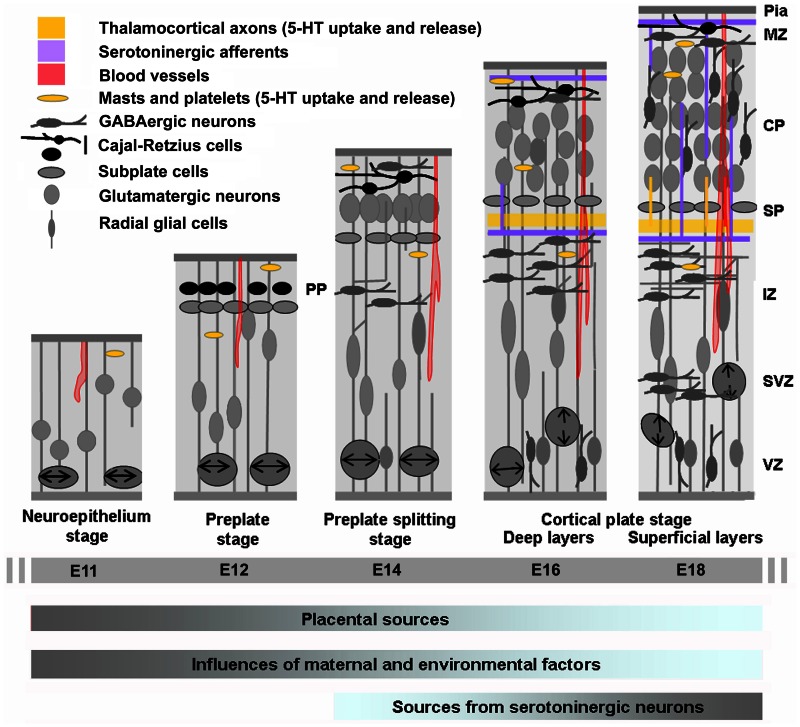
**Cortical development in relation to sources of 5-HT**. Cortical neurogenesis in the mouse neocortex occurs from embryonic day E10–11 (left) to E17 (right) begins with an intense proliferation of the progenitor cells located in the ventricular zone (VZ) of the subpallium and more ventrally of the pallium (not shown in the drawing). These populations of cells give rise to most of the GABAergic neurons (subpallium) and glutamatergic neurons and glial cells (pallium) of the cerebral cortex. Once generated, neurons migrate toward the pial surface and complete their differentiation in the cortical plate (CP). Glutamatergic neurons destined to populate the deeper layers of the cortex are generated and then migrate away form the VZ earlier than the neurons destined for progressively more superficial layers. GABAergic neurons arise from more ventral structure and migrate tangentially in the developing CP. On E13, the cerebral wall is bilaminar consisting of the VZ and overlying primitive plexiform layer. By E17–E20 the thickness of the overlying intermediate zone/with matter and developing cortical plate are at their maximum widths, with all neuronal cells having exited the cell cycle and migrated to their final laminar distribution within the developing cortex. At this stage GABAergic neurons enter the CP by radial migration. The cortical anlage is vascularized very early and carries platelets and mast cells that could provide 5-HT to the developing embryo. During the initial phase of cortical development 5-HT is mainly synthesized in the placenta while later on it is produced by serotonergic neurons of the embryo (gray is high and blue is low). IZ, intermediate zone; PPL, primordial plexiform layer; SP, subplate; SVZ, subventricular zone [Adapted from Uylings et al. (1990) and Corbin et al. (2008)].

Although 5-HT is likely to act as a trophic or instructive factor during early periods of cortical development, its sources have remained elusive. Evidence indicates that 5-HT is supplied to the developing cerebral cortex before 5-HT axons reach their targets or even before serotonergic neurons are generated. In line with this observation, 5-HT receptors are expressed in the rostral forebrain, craniofacial region, and peripheral region days before serotonergic axons enter these regions (Buznikov et al., 2001). Furthermore, *ex vivo* application of 5-HT or alteration of 5-HT levels during early embryonic stages can alter normal development of various embryonic structures before serotonergic neurons have innervated these structures (Lauder, 1988; Shuey et al., 1992; Moiseiwitsch and Lauder, 1995; Whitaker-Azmitia et al., 1996; Buznikov et al., 2001; Witaker-Azmitia, 2001). Recently, the placenta (that is of embryonic origin) has been identified as an important source of 5-HT for the developing embryo (Bonnin et al., 2011; Figure 2). Syncytiotrophoblastic cells of the placenta contain Tph1, AADC, and MAO (Grimsby et al., 1990; Shih et al., 1990), and convert tryptophan of maternal origin into 5-HT as soon as E10–E11 (Bonnin et al., 2011). Genetically modified mice in which 5-HT neurons fail to fully differentiate or to produce normal amounts of 5-HT levels do not display severe cortical defects when gestating in heterozygous dams with an almost unaltered serotonergic system, suggesting that sources of 5-HT independent of embryonic serotonergic neurons could be sufficient to permit normal cortical development. Examples include mice lacking the transcription factors Lmx1b (Smidt et al., 2000) or Pet-1 (Hendricks et al., 1999), in which all or 70–80% of 5-HT raphe neurons fail to develop, respectively, and in mice lacking Tph2 Alenina et al., 2009; Gutknecht et al., 2012; Migliarini et al., 2012. Further analysis revealed that Pet-1 knockout embryos developing in heterozygous dams have normal 5-HT levels before the closure of the brain-blood barrier (before E15; Daneman et al., 2010). In addition, SERT^+/−^ embryos developing in SERT^−/−^ or wild type dams had similar levels of 5-HT before E15 (Bonnin et al., 2011). Together, these results revealed that the placenta is an important source of 5-HT for the embryonic CNS before E15 but questioned the contribution of maternal 5-HT that was suspected in earlier studies (Shuey et al., 1992; Yavarone et al., 1993; Côté et al., 2003, 2007).

Outside the CNS, 5-HT is also synthesized in the periphery of the developing embryo. In particular, high levels of 5-HT are produced in the myenteric plexus (from E15 to E16), by enterochromaffin cells of the lining lumen of the digestive tract (from E18), by neuroepithelial cells of the respiratory tracts, by pinealocytes (from E11 to E12) and by parafollicular cells of the thyroid. After being released from 5-HT producing cells, 5-HT could be taken up by SERT expressing cells including platelets and mast cells (Jankovic, 1989; Zhuang et al., 1996) that become numerous around E12 in mice. These cells could cross the blood-brain barrier and transit across blood vessels that start to invade the developing cortex by E10–E11 in mice (Daneman et al., 2010). However, overall peripheral structures are thought to contribute only to a small proportion of cortical 5-HT during development.

In addition, sensory thalamic neurons projecting to primary sensory cortices (i.e., somatosensory, visual, auditory) transiently express SERT (E15–P15) and the vesicular monoamine transporter type 2 (VMAT2) that are respectively responsible for the uptake and packaging of 5-HT into synaptic vesicles (Cases et al., 1996, 1998; Vitalis et al., 1998; Lebrand et al., 1996, 1998; Gaspar et al., 2003; Vitalis and Parnavelas, 2003; Figure 2). While equipped with these transporters, thalamic neurons may release 5-HT in an activity-dependent fashion by transiently adopting a serotonergic phenotype even without expressing TPH or MAOs (Vitalis et al., 2002a). Interestingly, it has been suggested that TCAs could be implicated in the proliferation and migration of glutamatergic neurons, and it is thus possible that release of 5-HT by TCAs could contribute to the regulation of these processes (Kennedy and Dehay, 1997; Edgar and Price, 2001). Fate mapping of SERT-expressing cells in mice revealed that in addition to the thalamus, also the cortex, hippocampus, hypothalamus, and brainstem harbor neurons that transiently adopt a serotonergic phenotype (Narboux-Nême et al., 2008). Within the cortex, transient SERT expression starts between E15 and P0 and is confined to layers V and VI (infralimbic, prelimbic, and anterior cingulate cortex) or layers II, V, and VI (posterior cingulate and retrosplenial cortex). The role of 5-HT signaling by these neurons remains to be elucidated. However, because of the spatial and temporal aspects of this phenomenon, it is tempting to speculate that transient serotonergic neurons might influence cortical maturation and circuit formation.

## 5-HT receptors with specific attention to the 5-HT_3A_ and 5-HT_6_ subtypes

### Transduction pathways

At least 14 genes that encode for 5-HT receptors have been identified and cloned in the mammalian brain (Hoyer et al., 1994, 2002; Raymond et al., 2001; Hannon and Hoyer, 2008; Millan et al., 2008). In addition, alternative splicing and RNA editing add to the diversity of 5-HT receptors. With the exception of the 5-HT_3_ receptors, all 5-HT receptors are coupled to G-proteins, leading to a categorization into four groups according to their second messenger coupling pathways. The 5-HT_1_ and 5-HT_5_ receptors are coupled to Gi/Go proteins and exert their inhibitory effects on adenylate cyclase inhibiting cAMP formation. The 5-HT_2_ receptors are coupled to Gq proteins and stimulate phospholipase C to increase the hydrolysis of inositol phosphates and elevate intracellular Ca^2+^. The 5-HT_4,6,7_ receptors are coupled to Gs proteins and are positively linked to adenylate cyclase and increase cAMP formation. 5-HT_3_ (5-HT_3A_ and 5-HT_3B_) receptors belong to a family of ligand-gated ion channel receptors that include nicotinic acetylcholine receptors, GABA_A_ receptors, and glycine receptors and that are modulated by intracellular cyclic AMP (Hoyer et al., 1994). The 5-HT_3_ receptors respond to neurotransmitter release via direct (through the 5-HT_3_ receptor itself) or indirect (via the activation of the voltage-gated Ca^2+^ channels) increase of Ca^2+^ entry into the cell (reviewed in Chameau and van Hooft, 2006). 5-HT_3_ receptors are composed of five subunits, with the majority being homomers of 5-HT_3A_ receptors. Heteromeric 5-HT_3AB_ receptors have been observed in specific brain regions and display lower Ca^2+^ permeability than the homomeric 5-HT_3A_ receptors (Tecott et al., 1993; Morales and Bloom, 1997; Davies et al., 1999; Morales and Wang, 2002). Furthermore, the co-assembly of the 5-HT_3_ with the alpha4 subunit of the nicotinic acetylcholine has been reported to confer increased permeability to Ca^2+^ (Kriegler et al., 1999; Chameau and van Hooft, 2006).

### Expression patterns

**Figure 3:**
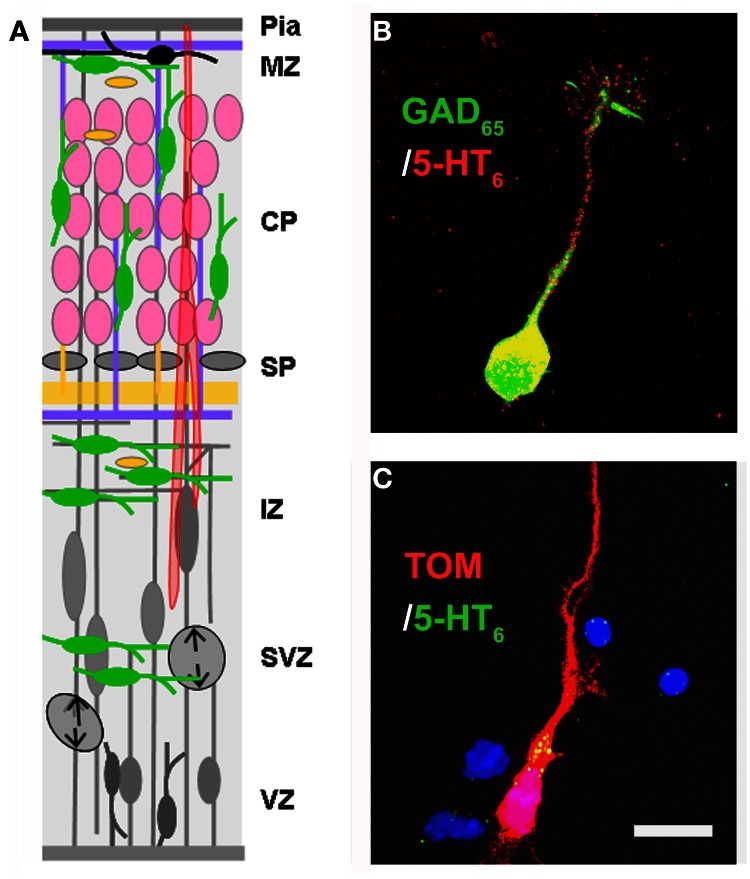
**Expression of the 5-HT_6_ receptor. (A)** Drawing shown in Figure 2 has been modified in order to depict the expression of 5-HT_6_ at E17–E18. At this stage 5-HT_6_ is expressed in developing interneurons (green), in differentiating pyramidal neurons (pink). **(B,C)** Expression of 5-HT_6_ in cell cultures (E17 + 1DIV) in GABAergic neurons expressing GAD_65_ (green) and in pyramidal neurons labeled with TOM after *in utero* electroporation performed at E14.5. Scale bar: 10 μm.

The expression of 5-HT receptors during cortical development is not yet fully characterized. However, the recent use of transgenic animals (i.e., carrying the GFP/YFP under the control of a specific 5-HTR promoter) and open *in situ* hybridization databases (i.e., Allen Brain Atlas) have started to provide valuable insights. For example, 5-HT_1A,F_ are expressed in neocortical proliferative zones in E14.5 rodent brain (Hillion et al., 1994; Bonnin et al., 2006) and the 5-HT_2B_ are expressed in the proliferative zones of the human occipital cortex (Lidov and Rakic, 1995). The 5-HT_1A,B,D_, 5-HT_2A_, 5-HT_2C_, and 5-HT_3A_, are expressed in specific subpopulations of postmitotic neurons (Hillion et al., 1994; Johnson and Heinemann, 1995; Tecott et al., 1993; Morales and Bloom, 1997; Bonnin et al., 2006; Chameau et al., 2009; Vucurovic et al., 2010; Tanaka et al., 2012), whereas the 5-HT_6_ is expressed in both migrating interneurons and pyramidal neurons (Riccio et al., 2011; Figure 3). Although a complete developmental time-course of 5-HT_6_ expression in the dorsal pallium is not available, 5-HT_6_ expression is detected in the developing rat brain as early as E12 and is maintained stable until adult age (Grimaldi et al., 1998). In adulthood, 5-HT_6_ receptors are expressed in layers II–VI of the rodent postnatal and mature cerebral cortex (Ward et al., 1995; Hamon et al., 1999; Gerard et al., 1997), and pyramidal neurons and glial cells of the human prefrontal cortex (Marazziti et al., 2013). Interestingly, human prefrontal cortex expression of the 5-HT_6_ receptor peaks in toddlers (Lambe et al., 2011).

**Figure 4:**
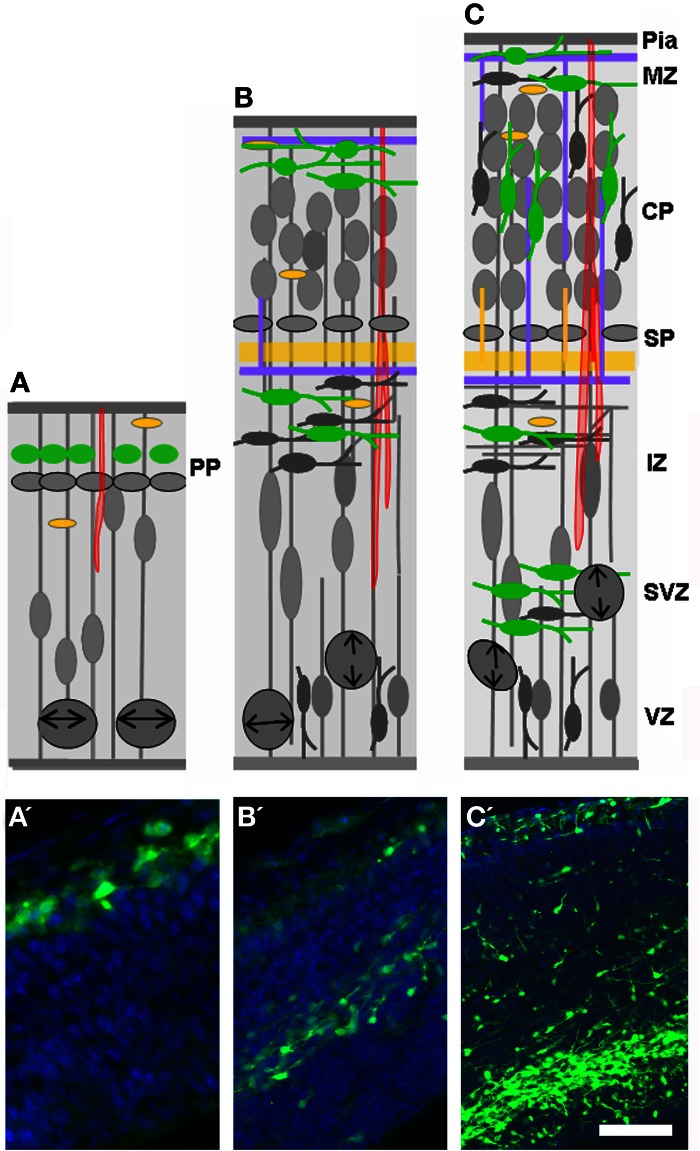
**Expression of the 5-HT_3A_ receptor. (A–C)** Drawings shown in Figure 2 have been modified in order to depict the expression of 5-HT_3A_ (green) during development. Note that 5-HT_3A_ is expressed by pioneer Cajal-Retzius cells of the marginal zone (MZ; **A**, E12) and by a subpopulation of late generated GABAergic neurons arising from the CGE (E14 and E16–E17). **(A′–C′)** Corresponding photomicrographes of the drawings presented in (**A–C**). Scale bar: **(A)** 20 μm; **(B)** 100 μm; **(C)** 120 μm.

The dynamic expression pattern of the 5-HT_3A_ receptor is recapitulated in Figure 4. In the mouse cortical anlage, 5-HT_3A_ is expressed as early as E12 in PP neurons expressing reelin (C-R cells) and/or GABA (Chameau et al., 2009; Vucurovic et al., 2010). During the period of intense production of GABAergic neurons, the 5-HT_3A_ is expressed by newly postmitotic (Tuj-1+) neurons located in the CGE and AEP/PO, where about 30% of cortical GABAergic neurons are generated (Lee et al., 2010; Vucurovic et al., 2010). Using homochronic *in utero* grafting in combination with a transgenic mouse line expressing GFP under the control of the 5-HT_3A_ promoter (5-HT_3A_:GFP animals) we have shown that this expression was protracted in two large subpopulations of cortical GABAergic neurons that could be distinguished based on their electrophysiological properties, molecular contents, and morphologies. The first one corresponded to multipolar interneurons expressing NPY and displaying late spiking and accommodating properties while the second one corresponded to small bipolar and doublet bouquet interneurons expressing VIP and displaying adapting and bursting properties (Vucurovic et al., 2010; Lee et al., 2010; Rudy et al., 2011; Vitalis and Rossier, 2011). During postnatal stages and decreasing with age 5-HT_3A_ receptors are also expressed in young neurons (doublecortin+ and/or CR+), which are generated in the SVZ and migrate toward the olfactory bulb and various cortical and subcortical regions (Inta et al., 2008; Riccio et al., 2012). In addition, we recently found that 5-HT_3A_ receptors are expressed during postnatal development (P0–21) in a pool of migrating interneurons, which are probably generated from local transient amplifying precursors within the white matter, ventral to the anterior cingulate cortex (Riccio et al., 2012).

## Impact of 5-HT imbalance on cortical circuit assembly

### 5-HT and cell proliferation

It has been postulated for some time that 5-HT regulates the proliferation of a wide variety of cell types including cortical neurons. Indeed, studies that pharmacologically or genetically deplete maternal and embryonic brain 5-HT levels or restrict tryptophan availability have found reduced embryonic brain size as a major consequence. Chronic pCholophenylalanin (PCPA)-treatment, which inhibits 5-HT synthesis, alters the proliferation of serotonergic target cells (i.e., the hippocampal field and cerebral cortex) when administrated daily to pregnant dams from E8 to E12 (Lauder and Krebs, 1978) or from E12 to E17 (Vitalis et al., 2007). Similar observations were made after reserpine-treatments that deplete 5-HT (Holson et al., 1994), or after lesions of serotonergic fibers such as those observed after high cocaine administration (Clarke et al., 1996). However, there are several drawbacks in these initial studies. For example, chronic treatments are likely to induce secondary alterations, which might be ultimately responsible for the effects observed. Another major problem is the selectivity of the neurotransmitter system affected. This is particularly problematic for reserpine-treatments that deplete all monoamines. Recently, the generation of transgenic models selectively targeting specific 5-HT-related genes in different neuronal populations have started to provide more specific insights. For instance mice deficient for *tph1* or *tph2* showed body weight reduction and delayed maturation of upper cortical layers (Côté et al., 2007; Alenina et al., 2009; Narboux-Neme et al., 2013). A 2 h pulse labeling experiment revealed that heterozygous embryos growing in null mutant tph1^−/−^ mice showed an ~30% reduction of BrdU-positive cells in the VZ when compared to tph1^−/−^ embryos growing in heterozygous mice (Côté et al., 2007). Together these studies suggest that 5-HT regulates the proliferation of neuronal precursors, but additional studies are needed to refine these initial observations and confirm this conclusion.

Initial *in vitro* studies have failed to show that 5-HT could modulate the proliferation of cortical progenitors (Dooley et al., 1997; Lavdas et al., 1997), as the proportion of cells that integrated BrdU was similar in untreated and treated cultures. However, since 5-HT had an anti-apoptotic effect the dilution of BrdU+ cells may have masked this proliferative effect. Furthermore, it was demonstrated that stimulation of the 5-HT_2_ and 5-HT_3_ had no effect on cortical neurogenesis (Dooley et al., 1997; Vitalis and Parnavelas, 2003). This is consistent with the fact that 5-HT_3A_ is not expressed in pallial and subpallial proliferative zones (Vucurovic et al., 2010). In contrast, the 5-HT_1A_ appears to mediate such a role. *In vivo*, PCPA-induced microcephaly is reversed after treatment with a 5-HT_1A_ agonist. Furthermore, in the adult rodent brain, 5-HT_1A_ promotes neurogenesis in the subgranular zone of the dentate gyrus (Brezum and Daszuta, 1999, 2000; Gould, 1999; Haring and Yan, 1999) and such a role has been postulated to be a key feature of antidepressant therapies (Guthrie and Gilula, 1989; Santarelli et al., 2003). Recently, the analysis of mice lacking MAOA and B, which display high 5-HT levels but normal dopamine and norepinephrine levels during embryonic and early postnatal development, revealed a specific reduction of symmetric divisions of intermediate precursor cells (Corbin et al., 2008) in the SVZ during late corticogenesis (E17.5; Cheng et al., 2010). This unexpected alteration was reverted after E14.5–E19.5 PCPA-treatment. In addition, neurosphere formation was modulated by 5-HT in a dose-dependent manner *in vitro*, with proliferative effects observed for concentration ranging from 10 to 100 ng/ml and inhibitory effects observed for higher concentration (1000 ng/ml). Interestingly, these inhibitory effects were associated with decreased 5-HT_1A_ labeling of neuronal precursors (Cheng et al., 2010). Together, these studies identified 5-HT_1A_ as a largely positive regulator of neuronal proliferation in embryonic and postnatal life. Hence, 5-HT might modulate cortical density through its proliferation-inducing action on progenitors.

Additional mechanisms exist through which 5-HT could potentially modulate proliferation and cortical density. 5-HT could be involved in modulating the length of the cell cycle or participate in progenitor cell death regulation. Interestingly, E12–E17 PCPA-treatment reduces the number of cells expressing Ki67 (a proliferation marker), promotes early GFAP expression, and impairs the normal development/organization of radial glial processes (Vitalis et al., 2007). In turn, early differentiation of RGCs could reduce cortical neurogenesis. Alternatively, hypo-5-HT induced microcephaly could be due to increased death of postmitotic neurons or neuronal progenitors. Indeed, 5-HT_2_ stimulation promotes the survival of glutamatergic neurons *in vitro* with a maximal effect observed for stages E16 and E18 in rats (Dooley et al., 1997), and 5-HT_1A_ stimulation increases neuroprotection in models of ischemia and protects neuronal cultures against serum withdrawal (Bielenberg and Burkhardt, 1990; Azmitia et al., 1995; Ahlemeyer et al., 2000). Furthermore, activation of 5-HT_2_ reverts increased apoptosis observed in VMAT2:KO mice, in which dopamine, norepinephrine, and serotonin are depleted (Stankovski et al., 2007). Such a role was also observed in mice lacking TrkB, the high affinity receptor for the brain-derived neurotrophins factor (BDNF) and neurotrophin 4, and in both cases 5-HT_2_ activation was able to normalize the caspase 3–9 cascades (Vitalis et al., 2002b; Stankovski et al., 2007).

During early development, 5-HT could also influence cortical proliferation through the modulation of gap junctions that coordinate cell-cell assembly (Guthrie and Gilula, 1989; Lo Turco and Kriegstein, 1995; Bittman et al., 1997). Interestingly, monoaminergic receptor activation modulates postnatal gap junction coupling in various brain regions including the developing neocortex, where regulation appears to occur at the level of connexin subunit phosphorylation (Röerig and Feller, 2000). Pharmacologic evidence suggests that 5-HT promotes uncoupling of gap junctions through 5-HT_2R_ stimulation (Röerig and Feller, 2000). However, to our knowledge, no study has investigated the action of 5-HT receptor modulation on gap junction coupling in the embryonic cortex.

### 5-HT and neuronal migration

**Figure 5:**
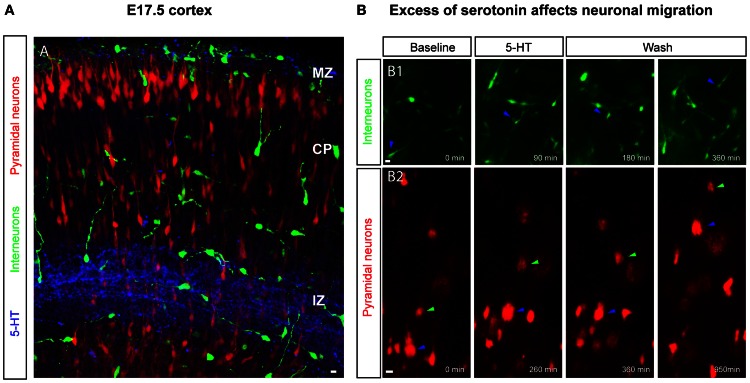
**5-HT levels impact the migration of GABAergic and glutamatergic neurons. (A)** 5-HT immunoreactivity is present in the marginal zone (MZ) and in the intermediate zone (IZ) at embryonic day E17.5, a time point when pyramidal neurons and cortical interneurons are migrating to constitute upper cortical layers. Upper-layer pyramidal neurons (in red) are labeled through electroporation of progenitor cells in the dorsal pallium at E14.5 or E16.5 with a Tomato-expressing plasmid and interneurons are labeled using GAD65-GFP or 5-HT_3A_-GFP transgenic mice. **(B)** Time-lapse imaging on cortical slices revealed that an excess of 5-HT and 5-HT_6_ manipulation affects the migration of cortical interneurons (B1) and pyramidal neurons (B2) in a reversible manner. Scale bar: 10 μm.

5-HT modulates the migration of various cell types and this effect is maintained across most phyla. For example, 5-HT acts as a permissive signal that triggers cell motility of mature lymphocytes in the vertebrate immune system (chick, fish, rodent; Khan and Deschaux, 1997; Boehme et al., 2004) and of microglial cells toward the central nervous system (Krabbe et al., 2012). In the non-vertebrate developing CNS a role for 5-HT in promoting-directed neuronal migration has been reported for *Caenorhabditis elegans* (Kindt et al., 2002). In the mammalian cortex, a role for 5-HT in regulating the migration of cortical neurons has emerged recently with studies focused on the late phase of corticogenesis. Using a pharmacological approach and cortical slices, high 5-HT levels have been shown to decrease the migratory speed of non-GABAergic and GABAergic neurons (Riccio et al., 2009, 2011; Figure 5). In cortical explants of E17.5 or P0 mouse brain, in which pyramidal neurons were labeled by *in utero* electroporation at E14.5 or E16.5 respectively, neuronal migration was analysed using video-microscopy in control condition or after acute bath application of 5-HT. This study revealed that acute application of high 5-HT concentration leads to a reversible decrease in the migration speed of glutamatergic neurons running in the IZ. Interestingly, SERT^−/−^ mice exhibit an abnormal distribution of pyramidal neurons in the most superficial regions of the CP at E19 (presumptive layers II–III) suggesting that 5-HT excess could lead to a delay in the migration of cortical pyramidal neurons *in vivo*. Furthermore, activation of the 5-HT_6_ receptor recapitulates these events: application of a specific 5-HT_6_ agonist to E17.5 or P0.5 cortical explants reduced the migratory speed of pyramidal neurons labeled at E14.5 or E16.5 respectively, suggesting that the 5-HT_6_ receptor is involved in regulating neuronal migration (Riccio et al., 2011). Similarly to non-GABAergic neurons, GABAergic neurons expressing GAD_65_ reversibly and in a dose-dependent manner decrease their migratory speed following acute high levels of 5-HT application *ex vivo* (Riccio et al., 2009). 5-HT also induced a retraction of the leading processes of GABAergic neurons migrating into the IZ and CP. RT-PCR performed on cells sorted by flow cytometry and obtained from E18.5 cortical slices of GAD_65_:GFP mice, revealed that these cells expressed the 5-HT_3A_ and the 5-HT_6_ receptors. Again, 5-HT_6_ agonist application mimicked 5-HT-induced effects on GABAergic neurons. Furthermore pharmacological manipulation of the cAMP-signaling pathway partially modulates the 5-HT_6_ mediated effects on cortical interneuron migration (Riccio et al., 2009). Interestingly, recent large-scale proteomic strategies have revealed that the 5-HT_6_ receptor binds to a large variety of signaling molecules that play a critical role during brain development including the mTOR pathway (Meffre et al., 2012). It is thus likely that the effects on migration elicited by the pharmacological manipulation of 5-HT_6_ receptors also involve these signaling pathways. Studies are currently underway to test this hypothesis.

It must be noted that the impact of 5-HT on the migration of cortical neurons was revealed using high doses of 5-HT. As in other cell types (Moiseiwitsch and Lauder, 1995), it is possible that 5-HT produces opposite effects on neuronal migration depending on the levels of extracellular 5-HT. In cortical explants maintained in a serum-free medium containing lower concentration of 5-HT than those used in experiments described above (5 uM), glutamatergic neurons reach their laminar location faster than in explants maintained in serum-free medium alone, suggesting that 5-HT may enhance the radial migration of these neurons (Lepore et al., 2001). Furthermore, decreasing 5-HT levels during development delayed or disrupted cortical migration suggesting 5-HT could also act as a positive drive on cortical migration (Stankovski et al., 2007; Vitalis et al., 2007). In animals treated with PCPA during the peak of migration (E12/E13 to E17 in rats), GABAergic neurons accumulated at the level of the SP and showed a marked deficit to integrate in the developing CP (Vitalis et al., 2007). Long-lasting consequences of E12–E17 PCPA-treatment lead to a marked reduction of CR- and CCK/VIP-positive GABAergic neurons, two neuronal populations that express the 5-HT_3A_ receptor (Férézou et al., 2007). Interestingly, mice lacking Tph2 also display reductions of selective GABAergic populations in limbic structures (Waider et al., 2013). 5-HT_3A_ is protractedly expressed by 30% of GABAergic neurons and it could be that this population is particularly sensitive to 5-HT depletion during corticogenesis. 5-HT_3A_ is associated with F-actin that decorates the tips of the of dendritic and axonal growth cones. Interestingly, pharmacological alteration of F-actin induced a modification in the distribution of 5-HT_3A_ (Emerit et al., 2002). In addition, 5-HT_3A_ mediates calcium entry into the cell (see above). Together these results suggest that 5-HT_3A_ activation could play a role in promoting the migration of cortical interneurons. Such a role is under investigation.

### 5-HT and differentiation

Lauder and Krebs were the first to report that 5-HT depletion delays neuronal maturation in areas normally receiving 5-HT afferents (Lauder and Krebs, 1978; Lauder, 1993). These investigators defined differentiation as the cessation of cell division measured by incorporation of ^3^H-thymidine. After these pioneering studies, numerous groups have shown that 5-HT can influence neuritic outgrowth in various phyla (such a role was intensively investigated in Aplysia) and in various regions of the CNS (Haydon et al., 1984, 1987; Whitaker-Azmitia et al., 1996; Lieske et al., 1999; Lotto et al., 1999; Kondoh et al., 2004; Fricker et al., 2005 and see below). Here we review the role for 5-HT on dendritic and axonal morphogenesis during cortical development.

#### 5-HT and dendritic maturation of cortical neurons

After termination of neuronal migration, cortical neuron subtypes differentiate at their specific laminar position and assemble into precise cortical circuits. During this process, projection neurons extend an elaborated dendritic arbor, which is contacted by the axons of different subtypes of excitatory neurons and inhibitory interneurons in a subdomain-specific manner. The molecular rules that govern the precise connectivity between different subtypes of inhibitory interneurons and excitatory projection neurons are largely unknown. In this context, reelin-secreting C-R cells have been identified as key regulators of cortical development, including neural migration, neural positioning, and dendritic arborization (Supèr et al., 2000; Soriano and del Rio, 2005; Lakatosova and Ostatnikova, 2012). C-R cells receive serotonergic projections with which they make transient synaptic contacts (Janusonis et al., 2004). Reelin secretion is regulated in part by the amount of brain 5-HT during late embryogenesis since 5-methoxytryptamine, a broad 5-HT receptor agonist, reduces reelin levels circulating in the blood at P0 (Janusonis et al., 2004). Reduced reelin levels in turn lead to malformation of microcolumns in the presubicular cortex of the P7 rat pups. Microcollumns are the basic microcircuit-units of the cortex (Jones, 2000; Mountcastle, 2003), and intriguingly are structurally abnormal in autism spectrum disorder (ASD). The 5-HT_3A_ is expressed by ~80% of C-R cells at P0 and its synaptic activation is sufficient to induce action-potential firing of C-R cells, suggesting that 5-HT_3A_ could play a role in regulating reelin release and dendritic development (Chameau et al., 2009). Indeed, developmental 5-HT_3A_ blockade induces a hypercomplexity of apical dendrites of layers II–III pyramidal neurons sparing the basal dendrites (Janusonis et al., 2004). In line with this finding, application of the N-terminal region of reelin rescued the dendritic phenotype of cortical pyramidal neurons in 5-HT_3A_:KO cortical slices, whereas reelin blockade leads to increased growth of apical dendrites (Chameau et al., 2009). These data suggest that, increased reelin secretion due to over-activation of the 5-HT_3A_ receptor would decrease growth of apical dendrites. This hypothesis was recently investigated *in vivo* using selective 5-HT re-uptake inhibitors (SSRI). Interestingly, fluoxetine administration from E8 to E18 decreases the dendritic basal and apical arbor complexity of layer II/III pyramidal neurons in the somatosensory cortex. This effect is specific to the developmental period as SSRI have opposite consequences at mature stages (Table 1 in Homberg et al., 2009). Furthermore, the effects of SSRIs on developing dendrites were abolished when administered to 5-HT_3A_:KO mice or after pharmacological blockade of the 5-HT_3A_ receptor (Chameau et al., 2009; Smit-Rigter et al., 2012). Moreover, 5-HT_3A_ signaling is responsible for the anxiety-like behaviors that are induced by prenatal fluoxetine treatment in wild type mice (Smit-Rigter et al., 2010). These results suggest that developmental excess of 5-HT increases reelin secretion by over-activating 5-HT_3A_ receptors expressed on C-R cells, consequently inhibiting dendritic growth of pyramidal neurons.

However, other 5-HT receptors may contribute to modulating the morphology of cortical neurons. The 5-HT_1A_ receptor for example is also known to modulate dendritic development (Sikich et al., 1990; Ferreira et al., 2010). Although its role has not been investigated in the cerebral cortex, several studies have clearly shown and dissected its role in the hippocampus. Indeed, mice lacking 5-HT_1A_ display increased dendritic arborization of CA1 pyramidal cells associated with cognitive impairments (Klemenhagen et al., 2006; Tsetsenis et al., 2007). Furthermore, the use of conditional expression of 5-HT_1A_ in mice otherwise lacking this receptor revealed that it is playing a critical role during the postnatal window corresponding to dendritic maturation of CA1 pyramidal neurons (Gross et al., 2002). During this time 5-HT_1A_ appears to limit dendritic growth cone retraction and extension by possibly remodeling actin filaments (Ferreira et al., 2010). As 5-HT_1A_ is strongly expressed in the developing CP (Figure 1 in Bonnin et al., 2006) such a role could also be expected for cortical neurons. Together these studies suggest that a fine tuning of 5-HT_1A_ activation may be required for appropriate dendritic maturation of cortical neurons. Finally, one should keep in mind that 5-HT also act as a trophic factor during development and 5-HT deficiency induces a reduction of dendritic arborization and complexity. Indeed, animals fed with low tryptophan diet (González-Burgos et al., 1996; Feria-Velasco et al., 2002) or depleted of 5-HT during the embryonic period (Vitalis et al., 2007) display cortical pyramidal neurons with decreased dendritic complexity and spine density. It is thus probable that 5-HT regulates dendritic maturation and spine density through different types of 5-HT receptors that remain to be identified.

#### 5-HT and axonal development within the cerebral cortex

**Figure 6:**
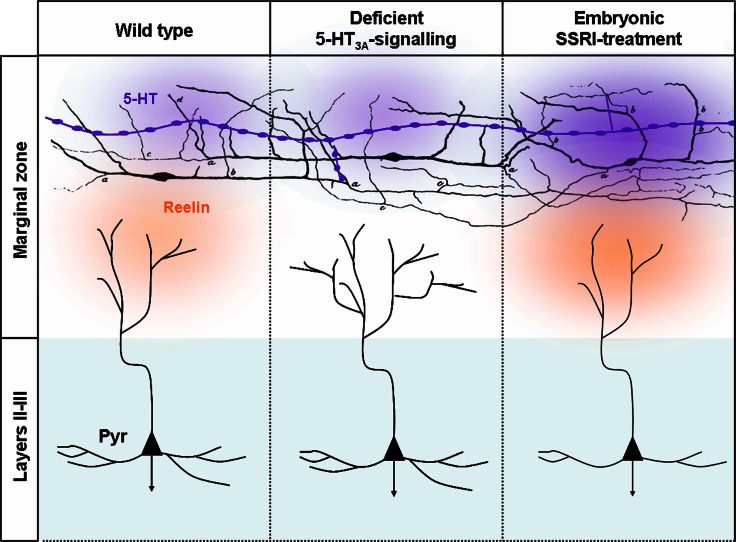
**Deficient 5-HT_3A_-signaling or embryonic SSRI-treatment alters the morphologies layers II–III pyramidal dendrites**. Serotonergic afferents (purple) containing large vesicles rich in 5-HT are located in the marginal zone and below the cortical plate that send branches toward MZ. 5-HT could be released from the entirety of the axonal length. In MZ, 5-HT fibers make synaptic contacts with C-R neurons that were initially drawn by Cajal (1891). In control conditions 5-HT activates 5-TH_3A_ receptors located on C-R neurons. 5-HT and the activation of the 5-HT_3A_ has been shown to induce reelin secretion that in turn modifies the morphology of apical dendrites by controlling the growth and sprouting of their arborization. In absence of 5-HT_3A_ receptor that could be observed in 5-HT_3A_-knockout mice or *ex vivo* following pharmacological blockade of the 5-HT_3A_ the morphology of apical dendrites become exuberant while basal dendrites that are far from the source of reelin are preserved. Following SSRI-treatment during embryogenesis excess extracellular 5-HT leads to an increased reelin secretion and to a reduction in the complexity of apical and basal dendrites.

**Figure 7:**
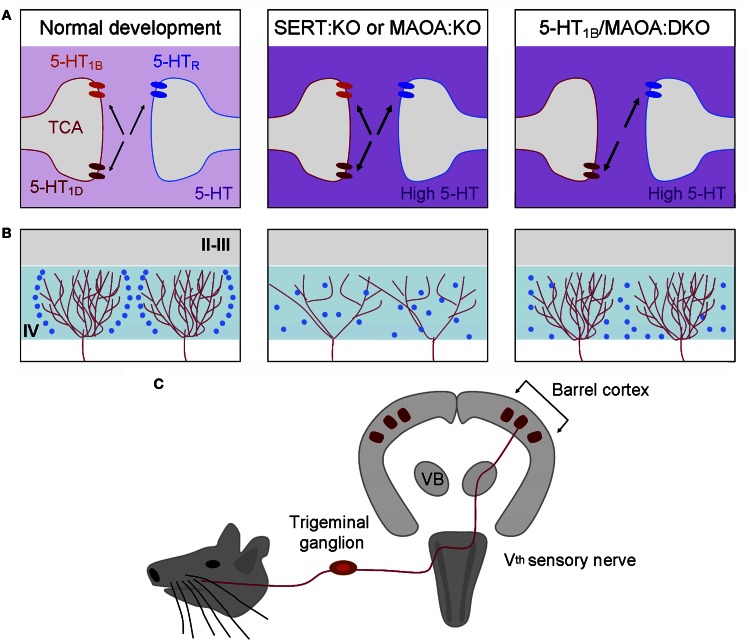
**The developmental organization of the somatosensory cortex depends on brain 5-HT levels**. The rodent barrel cortex is characterized by a one-to-one correspondence between the sensory system and its cortical projection area **(C)**. Each whisker on the rodent snout is somatotopically represented in the trigeminal nucleus, the ventrobasal thalamic nucleus (VB), and the primary somatosensory cortex (barrel). In the layer IV (IV) of the primary somatosensory cortex (barrel cortex), cortical barrels are constituted by regions of dense arborization of thalamocortical afferents (red) and by granular neurons (blue) that segregate around them. During normal development, SERT, 5-HT_1B_, and 5-HT_1D_ are expressed transiently by thalamocortical axons (left column). In SERT:KO or MAOA:KO mice displaying excessive extracellular 5-HT levels (deep purple) during development the organization of thalamocortical axons and the segregation of granular neurons are altered (middle column). 5-HT_1B,1D_ receptors are the direct targets of 5-HT excess. 5-HT_1B_ receptors act as regulators of thalamocortical development through the inhibition of glutamate release. Removing the 5-HT_1B_ in MAOA:KO mice is sufficient to restore a normal thalamocortical organization (right column). 5-HT_1B_:KO mice display normal barrel organization suggesting that 5-HT_1B_- and 5-HT_1D_-signaling are redundant and that 5-HT_1D_ is sufficient to maintain a normal organization of thalamocortical axons. However, granular neurons do not completely segregate suggesting that other 5-HT receptors, such as the 5-HT_2A_, could act on granular neurons and participate in the organization of cortical barrels. **(A)** Depicts what is occurring at the thalamocortical and granular interface. **(B)** Represent the organization of two adjacent barrels.

The first clear demonstration that 5-HT acts on cellular processes involved in the formation of cortical circuits comes from the work performed on the rodent somatosensory cortex (Figure 6). The serendipitous generation of a mouse displaying deficiency in the gene encoding for MAOA was at the starting point of these discoveries. These studies showed that excessive 5-HT amounts (nine-fold increase at P0) in the developing cortex induced an abnormal organization of TCAs growing in the layer IV of the primary somatosensory cortex (Cases et al., 1995, 1996; Figure 7). These alterations, that were later interpreted as an abnormal refining of TC axons, are due to a specific rise of 5-HT occurring during early postnatal development (P0–P4). Indeed, such alterations could be induced in wild type rodents by pharmacological inactivation of MAOA during this sensitive period (Vitalis et al., 1998).

In addition, pharmacological normalization of 5-HT levels in MAOA:KO mice by P0–P4 PCPA-treatment was sufficient to normalize the organization of S1 in MAOA:KO mice (Cases et al., 1996; Figure 7). Therefore, the first few days after birth represent the sensitive time-period for 5-HT effects on axonal segregation in the rodent barrel cortex. Later, it was shown that genetic SERT deficiency affected S1 organization similarly. The 5-HT 5-HT_1B_ and 5-HT_1D_ receptors, that are transiently expressed on TC axons during development, play a key role in this process, since the barrel cortex phenotype is rescued in SERT:KO and MAOA:KO mice that are also deficient for 5-HT_1B_ receptors (Persico et al., 2001; Salichon et al., 2001; Rebsam et al., 2002; van Kleef et al., 2012; Figure 7). The general model thus supports the view that increased extracellular levels of 5-HT lead to an over-activation of 5-HT_1B_ receptors expressed on TCAs. This increased 5-HT_1B_ signaling may inhibit glutamate release by TCAs and impair barrel cortex formation directly at presynaptic and indirectly at postsynaptic levels. Interestingly, 5-HT excess does not only impair S1 organization, since abnormal axonal patterning of TCAs was also observed in the primary visual cortex (Upton et al., 1999; Salichon et al., 2001). This intriguing role of 5-HT signaling during circuit formation may apply to all primary sensory cortices that are innervated by neurons transiently capable of 5-HT uptake (Hansson et al., 1998; Lebrand et al., 1998). Surprisingly, perinatal 5-HT deficiency induces only little changes on the organization of TCAs. Lowering brain 5-HT levels prenatally using PCPA or PCA only leads to a reduction of barrel field size (20% average) without altering its general organization (Bennett-Clarke et al., 1994; Osterheld-Haas et al., 1994; Narboux-Neme et al., 2013).

Although no evidence to date indicates that developmental excess of 5-HT during stages of embryonic developmental directly affects the patterning of TCAs, it has been shown that TCAs express 5-HT_1B_ and 5-HT_1D_ receptors at a time when TCAs are navigating from the subpallium toward the pallium (Bonnin et al., 2007). *In vivo* embryonic down-regulation of 5-HT_1B/C_ receptors in TCAs using *in utero* electroporation leads to abnormal TCA pathfinding indicating that 5-HT receptors are functional before birth and can regulate TCAs guidance at early stages of cortical development (Bonnin et al., 2007). Furthermore, it has been shown that 5-HT modifies the attractive vs. repulsive responsiveness of TCAs to netrin-1 (Bonnin et al., 2007), an important guidance molecule for TCAs. Given these findings, it is thus likely that developmental excess of 5-HT could also affect these earlier stages of thalamocortical pathfinding and lead to abnormal thalamocortical long-range wiring (Bonnin and Levitt, 2011; Bonnin et al., 2012).

## From rodent models to human pathology–translational considerations

**Figure 8:**
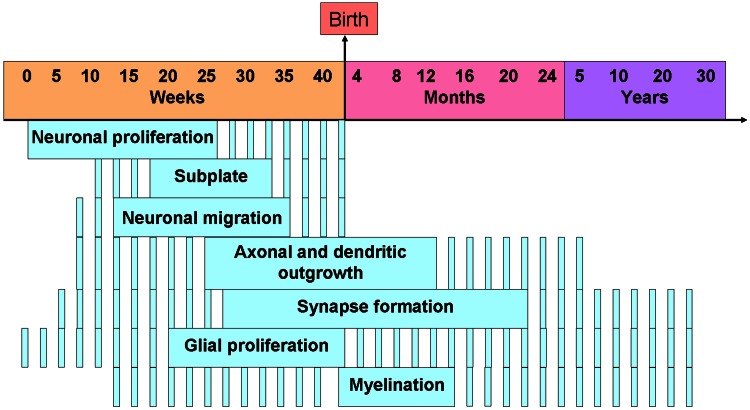
**Timing of the human developmental processes in the cerebral cortex**. Note that the dashed lines means that the process is active and plain lines means that the process is at its peak. Adapted from de Graaf-Peters and Hadders-Algra (2006).

The work reviewed reveals that developmental imbalance of 5-HT homeostasis or 5-HT receptor signaling has an impact on various processes involved in the formation of cortical circuits in rodents. Whether these developmental changes can also occur in humans remains largely unknown. The nature and severity of neocortical circuit alterations induced by 5-HT-related perturbations are likely to depend on a broad variety of factors including the timing of the insult. For instance, altered neuronal migration was observed during the late phase of rodent gestation, a developmental phase which corresponds to the second trimester in humans. In contrast, altered dendritic growth was observed largely during the first two postnatal weeks in rodents, a phase corresponding to the third trimester in humans. In this respect, we provide a rodent to human correspondence of cortical development in Figure 8 to ease comprehension.

One of the most clinically relevant situations leading to a developmental excess of 5-HT in humans is the exposure of the human fetus to SSRIs during pregnancy. SSRIs cross the placenta, reach the developing brain and are poorly metabolized by the fetus. Given the high incidence of mood disorders in pregnant women, prescription of SSRIs is frequent during this period. These drugs are considered relatively safe and beneficial during pregnancy, largely because they buffer the negative effects of maternal stress on the fetal-developing brain without causing major teratogenic effects. However, multiple negative effects of SSRI treatment during pregnancy have recently been identified, with the limitation that it is often difficult to control for confounding effects of maternal psychopathology. Ultrasonic investigation of human fetuses provides evidence that SSRIs taken during pregnancy alter the brain physiology starting as early as the beginning of second trimester (Mulder et al., 2011). Combined recordings of general motor activity, rapid eye movements, and fetal heart rate variability indicate that fetuses exposed to SSRIs during gestation have abnormal increases in motor movements during phases of non-REM sleep compared to fetuses from drug-free mothers with comparable levels of anxiety and depressive symptoms. Furthermore blood flow recordings at 36 weeks gestation in the middle cerebral artery were significantly decreased in fetuses exposed to SSRIs during gestation (Rurak et al., 2011). At birth, babies prenatally exposed to SSRIs display a wide range of neurobehavioral alterations, including lower APGAR scores, increased irritability, and blunted pain reactivity (Casper et al., 2003; Oberlander et al., 2005), as well as reduced fetal head growth (El Marroun et al., 2012). More recently prenatal antidepressants were shown to shift developmental milestones on infant speech perception tasks *in utero* and at 6 and 10 month of age (Weikum et al., 2012), suggesting a role for 5-HT in modulating critical time period maturation in humans. At later time-points, children exposed to SSRIs during pregnancy display increased internalizing behaviors (Oberlander et al., 2010) and decreased scores on psychomotor developmental scales (Casper et al., 2011). The most worrisome finding comes from a recent study reporting a two-fold increase in the risk for autism-spectrum disorders in children exposed to SSRIs during pregnancy (Croen et al., 2011). The risk appeared higher when exposure to SSRIs occurred during the second trimester and with higher dosage of SSRIs, suggesting deleterious effects on early neural circuit formation.

A second cause of excessive 5-HT-signaling in humans can be of genetic origin. The common 5-HT transporter-linked polymorphic region (SERTLPR) short (s) allele variant leads to decreased levels of SERT expression *in vitro* compared to the long (l) allele, and to a state of SERT hypofunction (Murphy and Lesch, 2008). This s-allele variant has been extensively investigated in the field of psychiatry and a large body of work in non-human primates and humans reveals that the hypofunctional s-allele interacts with early-life adversity to increase risk for a wide range of psychopathological traits. When exposed to high levels of maternal anxiety during pregnancy, 6 months old infants and children carrying the s-allele showed respectively higher levels of negative emotionality compared to l-allele carriers (Pluess et al., 2010) and increased scores of anxiety and depression (Oberlander et al., 2010). Finally an interaction between the s-allele and severe forms of adversity occurring later during childhood have been observed in many independent studies and lead to an increased risk for depressive symptoms in early adulthood (Karg et al., 2011). These findings indicate that the common hypofunctional s-allele is associated to an increased risk to broad spectrum of psychopathology in the presence of developmental adversity. The effect size of the s-allele is small and it is thus likely that the abnormal cortical circuit alterations observed in SERT deficient rodent models will only occur in humans in more severe forms of genetic or environmental SERT deficiency. In a clinical perspective, it is possible that only an accumulation of risk factors will lead to the cortical circuit alterations detected in rodents. For example, it is possible that these early life circuit alterations could emerge in fetuses carrying hypofunctional SERT variants and being exposed to SSRIs. Furthermore other risk alleles could interact with SERT deficiency to further increase the risk for neural circuit alterations. For instance, PTEN, a gene associated to ASDs (Levitt and Campbell, 2009) interacts with SERT haploinsufficiency to modify brain size and social behaviors in rodents (Page et al., 2009). Overall, these findings point to the general conclusion that various different clinical dimensions including autism, depression, and anxiety-related phenotypes are associated to conditions of SERT deficiency during development. Knowledge derived from animal studies is beginning to provide important insight into the developmental and cellular mechanisms that underlie these complex phenotypes. They support the general hypothesis that developmental excess of 5-HT can lead to early neural circuit alterations, which will act as an important vulnerability factor for a spectrum of psychiatric symptoms.

Rodent studies have revealed that the 5-HT_3A_ and the 5-HT_6_ receptors regulate cellular events involved in cortical circuit formation. However, their implication in determining vulnerability to human psychiatric disorders remains to be elucidated. Interestingly it has been reported that a 5-HT_3A_ genetic variant interacts with early-life adversity to increase risk for depressive symptoms and decrease fronto-limbic gray matter (Gatt et al., 2010). In addition this variant also interacts with polymorphisms in the brain-derived neurotrophic factor gene to predict emotion-elicted heart-rate, electroencephalogram asymmetry, and self-reported negativity bias (Gatt et al., 2010). These studies point to a potential developmental interaction between the 5-HT_3A_ receptor and early-life stress in mediating risk for mood disorders, confirming the intricate connection between early-life stress and the serotonergic systems. A role for the 5-HT_6_ receptor in determining risk for human psychiatric disorders still remains elusive. Human variants in the 5-HT_6_ receptor have initially been associated to an increased risk for schizophrenia but a recent meta-analyis reported negative findings (Kishi et al., 2012). Interestingly and in a developmental perspective, 5-HT_6_ antagonists have recently been shown to reverse cognitive deficits induced by early-life social isolation (Marsden et al., 2011). In two different developmental rat models of schizophrenia specifically neonatal phencyclidine and postweaning isolation, the mammalian target of rapamycin (mTOR) pathway was found to be persistently upregulated in the prefrontal cortex (Meffre et al., 2012). Interestingly it has been shown that 5-HT_6_ signaling acts on the mTOR pathway and that 5-HT_6_ antagonists injected in adulthood could reverse the cognitive defects induced by early-life insults and normalize mTOR signaling pathway modifications (Meffre et al., 2012). In a broader perspective pro-cognitive behavioral effects of 5-HT_6_ receptor antagonists have been observed in different types of animal models including socially isolated reared rats (Marsden et al., 2011). More specifically it has been shown that 5-HT_6_ receptor antagonists could reverse deficits in novel object discrimination induced by isolation rearing and that these procognitive effects could be linked to increased hippocampal-prefrontal cortex glutamatergic neurotransmission, further suggesting the relevance of the 5-HT_6_ receptor as a potential therapeutical target in cognitive deficits (Marsden et al., 2011).

## Perspectives

Data obtained in rodents and humans lead to the general hypothesis that genetic and environmental factors that influence 5-HT signaling during specific sensitive periods of development critically impact cellular events involved in the formation and maturation of cortical circuits. These various factors act in concert in predisposing to or protecting against cortical dysfunction. The central aspect of this conceptual framework is that type and timing of altered 5-HT signaling determine cortical circuit alterations and behavioral/cognitive consequences. Future studies will aim to focus on cell-type specific targets of 5-HT during development in order to gain a more precise understanding of the diversity of cellular events and receptors that are involved in cortical circuit formation. These studies should help us to better understand how 5-HT signaling during development can impinge on specific sets of neural circuits and how these circuit specific alterations are linked to the broad range of behavioral dimensions resulting from early-life 5-HT dysregulation.

### Conflict of interest statement

The authors declare that the research was conducted in the absence of any commercial or financial relationships that could be construed as a potential conflict of interest.
